# Factors Influencing Phenoconversion in CYP-Mediated Drug Metabolism: A Scoping Review

**DOI:** 10.3390/pharmacy14040108

**Published:** 2026-07-16

**Authors:** Sierra Scodellaro, Stefanie Triantafilou, Iris Cohn

**Affiliations:** 1 Division of Clinical Pharmacology and Toxicology, Department of Pediatrics, The Hospital for Sick Children, Toronto, ON M5G 1E8, Canada; sierra.scodellaro@sickkids.ca (S.S.);; 2 Program in Translational Medicine, The Hospital for Sick Children, Toronto, ON M5G 1E8, Canada; 3 Department of Pediatrics, Temerty Faculty of Medicine, University of Toronto, Toronto, ON M5G 1E8, Canada

**Keywords:** phenoconversion, pharmacogenetics, drug metabolism, precision medicine, clinical implementation

## Abstract

Pharmacogenetics (PGx) aims to optimize drug therapy by predicting medication response based on genetic variation in drug-metabolizing enzymes. However, observed drug metabolism may differ from genotype-predicted metabolizer status given external or acquired influences. This phenomenon is known as phenoconversion. As PGx testing becomes increasingly integrated into clinical care, understanding the contributing factors of phenoconversion is important for interpreting test results and guiding treatment. This scoping review aimed to categorize reported contributors of phenoconversion affecting CYP2D6, CYP2C19, and CYP3A4 metabolism. A structured literature search was conducted across Ovid MEDLINE^®^, Embase Classic + Embase, EBM Reviews, and Clarivate Web of Science. Records were screened using predefined inclusion and exclusion criteria, and data were extracted from eligible studies describing factors associated with metabolic activity inconsistent with genotype-predicted phenotypes. From 6008 records identified, 43 studies met the inclusion criteria. Reported contributors clustered into pharmacological factors, including drug–drug and drug–drug–gene interactions, clinical factors such as inflammation, physiological and demographic factors including pregnancy and age-related changes, and environmental influences such as smoking. Pharmacological contributors were most frequently studied, whereas environmental and demographic influences were less well characterized. These findings highlight the multifactorial nature of phenoconversion and underscore the need for further investigations to support phenoconversion integration into PGx-informed clinical decision-making.

## 1. Introduction

Pharmacogenetics (PGx) offers the potential to guide drug therapy by predicting an individual’s medication response based on genetic variation in drug-metabolizing enzymes [1]. However, observed clinical outcomes do not always align with genotype-based predictions. This discrepancy is often attributed to phenoconversion, a phenomenon in which external or acquired factors modify enzyme activity, resulting in a functional metabolic phenotype that differs from that predicted by genotype. Phenoconversion may be transient or sustained and can alter drug-metabolizing enzyme activity, leading to reduced, enhanced, or otherwise unpredictable metabolic function [2]. As PGx testing becomes increasingly integrated into clinical practice, understanding the factors that influence metabolic phenotype is essential for accurate interpretation of PGx results.

Phenoconversion has been most frequently described in relation to cytochrome (CYP) P450 enzymes, particularly CYP2D6, CYP2C19, and CYP3A4, which collectively metabolize a significant proportion of routinely used medications and are among the most clinically relevant enzymes that carry clinical PGx practice guidelines. Contributors to phenoconversion include co-medication use, comorbidities, inflammation, cancer, and lifestyle factors such as smoking or alcohol use [3]. The consequences can be clinically significant, leading to subtherapeutic drug exposure, increased toxicity, or therapeutic failure [4].

Despite these implications, phenoconversion remains relatively under studied, and evidence describing its contributing factors is dispersed across therapeutic areas and study designs. Evidence exploring the various non-genetic factors reported to influence phenoconversion across CYP2D6, CYP2C19, and CYP3A4 is generally limited. As the clinical implementation of PGx continues to expand, a clearer understanding of the factors that modify metabolic phenotype is needed. Accordingly, this scoping review aimed to systematically categorize reported factors influencing phenoconversion in CYP-mediated drug metabolism, identify patterns in the literature, and highlight opportunities for future research.

## 2. Materials and Methods

### 2.1. Identification of Eligible Studies

For this review, identification and selection of studies were performed according to the Population, Intervention, Comparison, and Outcome (PICO) framework [5]. Reporting was informed by the Preferred Reporting Items for Systematic Reviews and Meta-Analyses (PRISMA) guidelines (Table S1) [6]. The primary objective was to map the breadth and nature of evidence related to factors influencing phenoconversion rather than to evaluate study quality or perform quantitative synthesis. With that in mind, studies that identified factors of phenoconversion were the primary target of the literature search. A scoping review approach was utilized given the heterogeneity of study designs and outcome measures.

A structured search strategy was applied across four electronic databases: Ovid MEDLINE^®^, Embase Classic + Embase, EBM Reviews, and Clarivate Web of Science. Search terms included “phenoconversion” and related synonyms or descriptions, combined with terms representing co-medication, comorbidities, physiological and environmental factors, and CYP2D6, CYP2C19, and CYP3A4 enzyme activity. Results were limited to English-language publications between 2020 and 2024. The full search strategy is provided in Appendix A.

Identified records were initially screened by title and abstract, excluding commentaries, editorials, narrative reviews, letters without original data, conference abstracts, and non-English publications. The full texts of the remaining studies were assessed for eligibility. For the purpose of this study, phenoconversion was defined as a discrepancy between genotype-predicted metabolizer status and observed metabolic phenotype attributable to non-genetic factors. Records must have examined the influence of one or more non-genetic factors on CYP-mediated drug metabolism and must explicitly document that genotyping of the study population was conducted. Additionally, the genotype-predicted metabolizer statuses must have been reported, along with any relevant data on observed metabolic phenotype, such as therapeutic drug monitoring data. Studies focusing exclusively on the effects of co-medications for a single genotype were excluded, as phenoconversion may differ across genotypes. Title and abstract screening, full text review and data extraction were each performed independently by two reviewers using Covidence© software (Veritas Health Innovation, Melbourne, Australia. Available at www.covidence.org (accessed on 13 August 2025) to manage records, remove duplicates, and track inclusion/exclusion decisions. Discrepancies were resolved through discussion.

### 2.2. Data Extraction

Data was extracted pertaining to study characteristics, enzymes evaluated, and reported contributors to phenoconversion. The reported contributors to phenoconversion were grouped into thematic domains based on emerging patterns in the data after extraction and review. This process resulted in four domains into which all included studies were grouped: pharmacological, clinical, physiological and demographic, and environmental. For studies in which multiple CYP enzymes were described, data was extracted and categorized based on each individual enzyme of interest when enzyme-specific results were available. Metabolizer phenotype assignment was standardized according to current guidelines; for instance, subjects labeled as extensive metabolizers (EM) in the original studies were classified as normal metabolizers (NM), and heterozygous NMs (one wild-type allele and one reduced function allele) were grouped as intermediate metabolizers (IM).

## 3. Results

### 3.1. Study Selection

The database search identified 6008 records across Ovid MEDLINE^®^, Embase Classic + Embase, EBM Reviews, and Clarivate Web of Science. After the removal of 2530 duplicate records, 3478 studies remained for title and abstract screening. Of these, 3305 records were excluded based on the predefined eligibility criteria.

**Figure 1:**
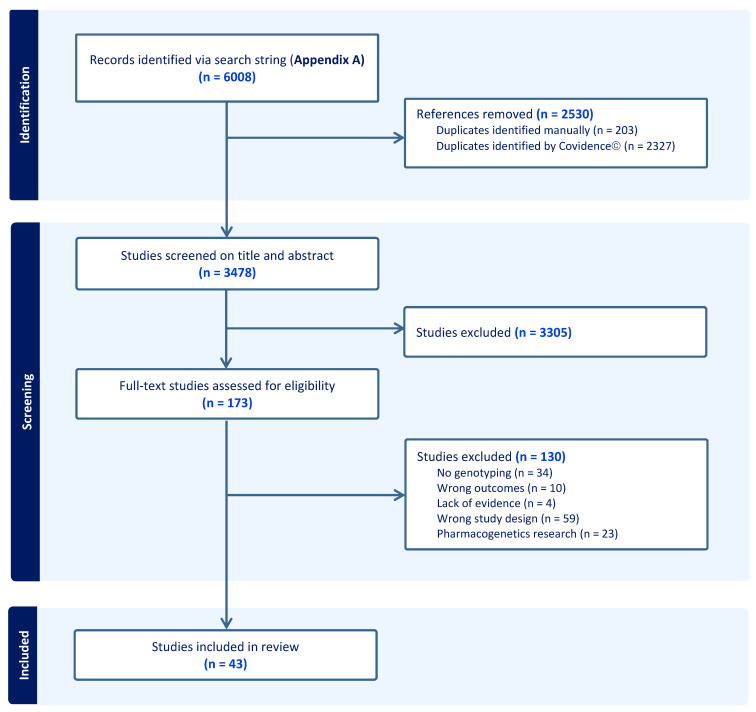
PRISMA diagram of the study retrieval and review process.

A total of 173 articles were assessed for full-text eligibility, of which 43 studies met the inclusion criteria and were included in the final synthesis. Reasons for exclusion at the full-text stage included lack of primary data, absence of genotype–phenotype comparison, failure to report specific factors influencing CYP-mediated drug metabolism, or simply providing PGx variant associations. The study selection process is summarized in the PRISMA flow diagram (Figure 1).

### 3.2. Characteristics of Included Studies

The 43 included studies examined factors influencing phenoconversion, primarily in relation to CYP2D6, CYP2C19, and CYP3A4 enzyme systems. Most studies were observational clinical investigations that evaluated drug metabolism using pharmacokinetic measurements or therapeutic drug monitoring. Fewer studies explored mechanistic or experimental models of phenoconversion. The characteristics of the included studies are summarized in Table 1.

Across the included literature, contributors to phenoconversion were grouped into four broad domains identified during data extraction: pharmacological factors (27/43), clinical factors (9/43), physiological and demographic factors (7/43), and environmental influences (1/43). Pharmacological factors were the most frequently investigated contributors, while environmental influences were less commonly observed.

### 3.3. Contributors to Phenoconversion

#### 3.3.1. Pharmacological Factors

Pharmacological factors, particularly drug–drug interactions and drug–drug–gene interactions, were the most frequently reported drivers of phenoconversion. Many studies described the impact of concomitant medications that inhibit or induce CYP enzyme activity, thereby altering the functional metabolizer phenotype.

Strong or moderate CYP inhibitors were frequently reported to convert individuals with normal or intermediate metabolizer genotypes into phenotypic poor metabolizers. Commonly implicated medications included antidepressants, antifungal agents, immunosuppressants, and antiepileptics. These interactions were most frequently described for CYP2D6 and CYP2C19 enzymes.

#### 3.3.2. Clinical Factors

Clinical conditions such as systemic inflammation, infection, and malignancy were also reported to influence CYP enzyme activity. Several studies suggested that inflammatory processes may suppress enzyme expression through cytokine-mediated mechanisms, resulting in reduced metabolic capacity.

In critically ill populations and individuals with significant comorbidities, altered drug metabolism was observed despite genotype-predicted metabolizer status, suggesting a role for disease-related phenoconversion. Overall, the evidence observed in this domain showed more heterogeneity and was less frequently quantified compared to pharmacological contributors.

#### 3.3.3. Physiological and Demographic Factors

Physiological and demographic influences included pregnancy, age-related changes in metabolic capacity, and oral contraceptive use. Pregnancy-related alterations in hepatic enzyme activity were reported to influence CYP3A4 and CYP2D6 metabolism.

Age-dependent changes were also described, particularly in pediatric populations where enzyme maturation may alter metabolic phenotype. Similarly, age-related decline in metabolic activity has been suggested in older populations. The evidence in this domain was primarily observational and carried limited consistency across study populations.

#### 3.3.4. Environmental Factors

Environmental exposures were less frequently observed but included factors such as smoking, alcohol use, and dietary influences. Smoking-related enzyme induction was described in several studies and was associated with altered CYP activity. However, environmental contributors were found to be the least studied and generally less well characterized compared to pharmacological or clinical factors.

## 4. Discussion

This scoping review highlights the multifactorial nature of phenoconversion and underscores the complexity of predicting drug metabolism based solely on genotype. While pharmacological factors, particularly drug–drug interactions, were the most frequently reported contributors, other influences such as comorbidities, different physiological states, and lifestyle factors received comparatively less attention. Across the included studies, pharmacological interactions involving CYP inhibitors or inducers were consistently identified as drivers of phenoconversion, reflecting the well-established impact of co-medication use on enzyme activity. In contrast, clinical and environmental influences, including inflammation, pregnancy, aging, and lifestyle exposures, were less frequently examined despite their potential in altering metabolic function. The limited identification of environmental contributors specifically, which was represented by only one included study, may reflect a genuine gap in current phenoconversion literature.

A notable finding across the literature was the lack of methodological consistency in how phenoconversion is defined and evaluated. Few studies applied standardized criteria for identifying phenoconversion, and approaches to assessing metabolic activity varied widely, including therapeutic drug monitoring, pharmacokinetic sampling, and indirect measures of enzyme function. Additionally, study designs and sampling timepoints differed substantially across investigations. This heterogeneity limits cross-study comparison and complicates efforts to quantify the magnitude and clinical relevance of phenoconversion across populations and therapeutic contexts.

Previously published reviews have described phenoconversion and summarized various key contributing factors within CYP P450 metabolism. Our findings, consistent with these reports, confirm the major role of pharmacological interactions, particularly CYP inhibition and induction, in contributing to phenoconversion. However, this scoping review builds on prior work by providing a structured categorization of contributing factors by systematically grouping studies across four main domains: pharmacological, clinical, physiological and demographic, and environmental. This approach not only highlights well-established pharmacological drivers but also the relative gap in evidence across other domains, such as physiological and demographic, and environmental.

The clinical implications of phenoconversion are increasingly recognized. Discrepancies between genotype-predicted metabolizer status and observed drug response are frequently encountered in clinical practice, and phenoconversion may provide an important explanation for this variability [50]. For example, concomitant use of CYP2D6 inhibitors, including certain antidepressants, can functionally convert normal metabolizers into poor metabolizers, resulting in altered drug exposure and therapeutic response. Similarly, inflammatory states, commonly observed in oncology, critical illness, and infectious disease, have been associated with cytokine-mediated suppression of CYP enzyme expression, which may contribute to unexpected drug toxicity or treatment failure. These findings highlight how dynamic physiological and pharmacological factors can modify metabolic phenotype in ways that are not captured by genotype alone.

As PGx testing becomes increasingly integrated into clinical practice, failure to account for phenoconversion may limit the predictive value of genotype-guided prescribing. Many current PGx implementation strategies rely primarily on static genetic information. However, the findings of this scoping review suggest that drug metabolism is influenced by a range of dynamic clinical factors that may fluctuate over time. As such, PGx results should be interpreted beyond genotype data alone. In clinical practice, this may look like reviewing concomitant medications that a patient may be taking, assessing inflammatory or disease states more thoroughly, and considering physiological factors such as pregnancy or age when interpreting PGx test results

Incorporating phenoconversion considerations into clinical decision support systems may also improve the accuracy of phenotype prediction and support more individualized dosing strategies, particularly in settings characterized by polypharmacy or complex comorbidities. For example, clinical decision support tools built within the electronic health record could integrate PGx results with real-time clinical data, such as co-medication use, disease states, inflammatory biomarkers and physiological factors to identify patients who may be most at risk for phenoconversion. Automated alerts could then prompt health care providers to reassess medication selection at the time of ordering. Despite this potential, limited systematic investigation and lack of standardized methodologies currently limit the integration of phenoconversion into routine clinical workflows [51].

Future research should focus on developing standardized definitions and methodological frameworks to evaluate phenoconversion. Consensus criteria for identifying phenoconversion, along with validated approaches for measuring metabolic activity, would facilitate more consistent reporting and enable comparisons across studies. Development of these standards will most likely necessitate collaboration among PGx consortia, researchers, clinicians, and regulatory stakeholders. Integrating these recommendations into PGx guidelines and clinical decision support systems would not only promote consistent implementation across healthcare settings, but also have the added benefit of facilitating international collaboration.

Prospective studies examining the clinical impact of phenoconversion on drug response and patient outcomes are also needed. Particular attention should be directed toward populations and therapeutic areas where dynamic physiological changes and complex medication regimens are common, including pediatric populations, transplantation, oncology, and critical care. Integration of real-time clinical data, such as medication exposure, inflammatory biomarkers, and physiological parameters, may further enable dynamic adjustment of metabolizer status and support more precise pharmacotherapy.

### Limitations

This review has several limitations. Although multiple databases were searched and dual screening was performed, the search was limited to English-language publications, which may have resulted in the exclusion of relevant studies. Additionally, given the limited number of studies examining environmental contributors to phenoconversion, this may reflect publication bias, which highlights gaps in available literature and the underreporting of relevant exposures impacting phenoconversion. For this scoping review, no formal protocol was prospectively registered. As a scoping review, this study aimed to map the breadth of existing evidence rather than to formally assess study quality or risk of bias. Heterogeneity in study design, phenoconversion definitions, and metabolic assessment methods further limited direct comparison across studies. Furthermore, the diversity of study populations and therapeutic areas represented in the included literature may limit the generalizability of these findings to specific patient populations or healthcare systems. While many contributors to phenoconversion are likely broadly applicable based on the included studies, the clinical impact may vary. These factors should be considered when interpreting the findings of this review.

## 5. Conclusions

Phenoconversion represents a clinically significant but under-recognized contributor to variability in drug response. This scoping review of 43 studies highlights the diverse factors that influence metabolic phenotype, including pharmacological interactions, comorbidities, physiological states, and lifestyle exposures. While pharmacological contributors are well characterized, non-pharmacological and environmental factors remain underexplored, and methodological heterogeneity limits cross-study comparability. Greater attention to standardized definitions, consistent assessment methods, and prospective evaluation is needed to better characterize phenoconversion and its impact on clinical outcomes. Integrating phenoconversion considerations into pharmacogenetic implementation and clinical decision support has the potential to enhance the precision of individualized therapy, particularly in populations with complex medication regimens or dynamic physiological states. Addressing these gaps will be essential to fully realize the promise of genotype-informed prescribing and to optimize patient-centered pharmacotherapy.

## Figures and Tables

**Table 1:** Reported contributors of phenoconversion.

Domain	Example Factors	Author (Year)	Reference
Pharmacological	Co-medication	Whitledge, J.D. et al., 2023	[7]
		Rodieux, F. et al., 2023	[8]
		Lenoir, C. et al., 2022	[9]
		Gloor, Y. et al., 2022	[10]
		Fekete, F. et al., 2021	[11]
		de Jong, L.M. et al., 2023	[12]
		Biswas, M. et al., 2021	[13]
		Bianconi, G. et al., 2022	[14]
		Bag, S. & Pradhan, S.K., 2023	[15]
		Chetty, M. et al., 2021	[16]
	Drug-drug interactions	Stöllberger, C. et al., 2023	[17]
		Russell, J. et al., 2023	[18]
		Patel, J. et al., 2024	[19]
		Kuhlmann, I. et al., 2021	[20]
		Del Toro-Pagán, N.M. et al., 2022	[21]
		Scherf-Clavel, M. et al., 2024	[22]
		Scherf-Clavel, M. et al., 2023	[23]
		Drevin, G. et al., 2021	[24]
		Giorgetti, A. et al., 2023	[25]
	Drug–drug–gene interactions	Nanan, C.J. et al., 2022	[26]
		Muhn, S. et al., 2022	[27]
		Kiss, A. et al., 2020	[28]
		Hole, K. et al., 2021	[29]
		Del Toro-Pagán, N.M. et al., 2021	[30]
		Hahn, M. & Roll, S.C. et al., 2021	[31]
		Nahid, N.A. & Johnson, J.A. et al., 2022	[32]
		Nakad, Z. & Saab, Y. et al., 2024	[33]
Clinical	Inflammation	Stanke-Labesque, F. et al., 2020	[34]
		Neyshaburinezhad, N. et al., 2023	[35]
		Li, Y. et al., 2024	[36]
		Ghasim, H. et al., 2023	[37]
		Deodhar, M. et al., 2021	[38]
		Bolcato, L. et al., 2021	[39]
		Aiuchi, N. et al., 2022	[40]
	Disease–drug interaction	Helsby, N. et al., 2021	[41]
		Rasool, S. et al., 2021	[42]
Physiological and demographic	Pregnancy	Gausi, K. et al., 2021	[43]
		Eniayewu, O. et al., 2023	[44]
	Oral contraception	Kuhn, A.K. et al., 2024	[45]
	Dietary supplementation	Matura, J.M. et al., 2022	[46]
	Age-dependent phenoconversion	Du, Y.X. et al., 2024	[47]
		Chen, J. et al., 2022	[48]
Environmental	Smoking	Lesche, D. et al., 2020	[49]

## Data Availability

No new data were created or analyzed in this study. Data sharing is not applicable to this article.

## Supplementary Materials

The following supporting information can be downloaded at: https://www.mdpi.com/article/10.3390/pharmacy14040108/s1, Table S1: Preferred Reporting Items for Systematic reviews and Meta-Analyses extension for Scoping Reviews (PRISMA-ScR) Checklist. Reference [52] is cited in the Supplementary Materials.

## Abbreviations

The following abbreviations are used in this manuscript:

PGx	Pharmacogenetics
PICO	Population, Intervention, Comparison, and Outcome framework
PRISMA	Preferred Reporting Items for Systematic reviews and Meta-Analyses

## Appendix A

**Table A1:** Ovid MEDLINE(R) Epub Ahead of Print and In-Process, In-Data-Review & Other Non-Indexed Citations and Daily (1946–1 July 2024).

#	Searches	Results
1	exp “Cytochrome P-450 Enzyme System”/or Cytochrome P-450 CYP2D6/ge, mt or “Cytochrome P-450 CYP3A”/ge or “Cytochrome P-450 CYP2B6”/ge or “Cytochrome P-450 CYP2C19”/	93,581
2	(“Cytochrome P-450*” or “Cytochrome P450*” or “25-Hydroxyvitamin D3 1-alpha-Hydroxylase” or “Aniline Hydroxylase” or “Aromatase” or “Aryl Hydrocarbon Hydroxylases” or “UDP-galactose transporter Benzopyrene Hydroxylase” or “Camphor 5-Monooxygenase” or “Cholestanetriol 26-Monooxygenase” or “Cholesterol 24-Hydroxylase” or “Cholesterol 7-alpha-Hydroxylase” or “Cholesterol Side-Chain Cleavage Enzyme” or “CYP11B2” or “CYP1A1” or “CYP1A2” or “CYP1B1” or “CYP2A6” or “CYP2B1” or “CYP2B6” or “CYP2C19” or “CYP2C9” or “CYP2C8” or “CYP2D6” or “CYP2E1” or “CYP3A” or “CYP4A” or “Limonene Hydroxylases” or “Retinoic Acid 4-Hydroxylase” or “Steroid 11-beta-Hydroxylase” or “Steroid 12-alpha-Hydroxylase” or “Steroid 16-alpha-Hydroxylase” or “Steroid 17-alpha-Hydroxylase” or “Steroid 21-Hydroxylase” or “Steroid Hydroxylases” or “Sterol 14-Demethylase” or “Trans-Cinnamate 4-Monooxygenase” or “Vitamin D3 24-Hydroxylase” or “drug metaboli?ing enzyme?” or “Cytochrome Drug-Metaboli?ing Enzyme?” or multigene* or multi-gene*).tw,kf.	127,488
3	1 or 2	156,115
4	“Dose-Response Relationship, Drug”/or “Precision Medicine”/or (“precision medicine*” or “personali?ed medicine*” or “Individuali?ed Medicine*” or Theranostic? or “Predictive Medicine*” or “Drug Dose-Response Relationship?” or (treatment* adj2 respon*)).tw,kf.	597,282
5	(interact* and (“drug-gene” or “drug-drug-gene” or “drug-gene-gene” or EADGI? or “drug-g-gene” or Pharmacogenomic or Pharmaco-genomic? or pharmacogenetic? or pharmaco-genetic? or DDI? or DDGI?)).tw,kf.	6568
6	(“phenoconversion” or phenoconver* or “pheno?conver*” or ((genotyp* or phenotyp* or extensive* or poor or intermediate or ultrarapid or ultra-rapid or rapid) and metaboli?er*)).tw,kf.	4893
7	or/4–6	607,345
8	“Comorbidity”/or (Comorbid* or “Co-morbidity” or Co-morbid* or Multimorbid* or Multi-morbid*).tw,kf.	362,438
9	exp “Cytokines”/or (“cytokine?” or chemokine?).tw,kf.	1,019,280
10	“Interleukin-4”/or “Interleukin-6”/or “Interleukin-13”/or (“Interleukin-4” or “B Cell Stimulatory Factor-1” or “B-Cell Growth Factor-1” or “B-Cell Proliferating Factor” or BCGF-1 or Binetrakin or BSF-1 or IL-4 or IL4 or “Mast Cell Growth Factor-2” or MCGF-2 or “Interleukin-6” or “B Cell Stimulatory Factor-2” or “B-Cell Differentiation Factor-2” or BSF-2 or “Hybridoma Growth Factor” or “IFN-beta 2” or “Plasmacytoma Growth Factor” or “Hepatocyte Stimulating Factor” or MGI-2 or “Myeloid Differentiation-Inducing Protein” or “B-Cell Differentiation Factor” or IL-6 or “Interferon beta-2” or IL6 or “Interleukin-13” or IL13 or IL-13).tw,kf.	259,408
11	(“adverse effect?” or “insufficient response?” or “pharmacokinetic?” or (drug adj3 (ineffective* or interaction? or efficac* or safe*)) or “serum concentration?” or polymorphism* or “clinical factor?” or (p?ediatric adj3 (tolerab* or consideration?))).tw,kf.	854,584
12	*”Antineoplastic Agents”/ae, me or (((Antineoplastic or Antitumo?r or Anti-neoplastic or Anti-tumo?r or Anticancer or anti-cancer or Chemotherap* or cancer) adj2 (drug* or therap* or treat* or agent?)) or Antineoplastic? or chemotreat* or chemotherap*).tw,kf.	861,557
13	“Antineoplastic Combined Chemotherapy Protocols”/or *”Drug Therapy, Combination”/or (“Antineoplastic Combined Chemotherapy Protocol?” or “Combined Antineoplastic Agent?” or “Anticancer Drug Combination?” or “Antineoplastic Combined Chemotherapy Regimen?” or “Antineoplastic Chemotherapy Protocol?” or “Cancer Chemotherapy Protocol?” or “Combination Chemotherap*” or “Drug Polytherap*” or “Combination Drug Therap*” or “Polychemotherap*” or “Poly-chemotherap” or comedicat* or “co-medicat*” or (concomitant adj (medication? or drug?)) or (medication adj manag*) or ((non-competitive or anti-competitive) adj3 inhibition)).tw,kf.	194,967
14	*”Medical oncology”/or neoplasms/or (cancer* or neoplas* or tumo?r* or oncolog*).tw,kf.	3,946,042
15	or/8–14	5,874,853
16	3 and 7 and 15	8535
17	limit 16 to (english language and yr = “2020 − Current”)	1878
18	remove duplicates from 17	1872

**Table A2:** Ovid Embase Classic + Embase <1947 to 2024 Week 26>.

#	Searches	Results
1	“cytochrome P450”/or “cytochrome P450 2D6”/or “cytochrome P450 3A”/or “Cytochrome P-450 2B6”/or “Cytochrome P-450 2C19”/	84,177
2	(“Cytochrome P-450*” or “Cytochrome P450*” or “25-Hydroxyvitamin D3 1-alpha-Hydroxylase” or “Aniline Hydroxylase” or “Aromatase” or “Aryl Hydrocarbon Hydroxylases” or “UDP-galactose transporter Benzopyrene Hydroxylase” or “Camphor 5-Monooxygenase” or “Cholestanetriol 26-Monooxygenase” or “Cholesterol 24-Hydroxylase” or “Cholesterol 7-alpha-Hydroxylase” or “Cholesterol Side-Chain Cleavage Enzyme” or “CYP11B2” or “CYP1A1” or “CYP1A2” or “CYP1B1” or “CYP2A6” or “CYP2B1” or “CYP2B6” or “CYP2C19” or “CYP2C9” or “CYP2C8” or “CYP2D6” or “CYP2E1” or “CYP3A” or “CYP4A” or “Limonene Hydroxylases” or “Retinoic Acid 4-Hydroxylase” or “Steroid 11-beta-Hydroxylase” or “Steroid 12-alpha-Hydroxylase” or “Steroid 16-alpha-Hydroxylase” or “Steroid 17-alpha-Hydroxylase” or “Steroid 21-Hydroxylase” or “Steroid Hydroxylases” or “Sterol 14-Demethylase” or “Trans-Cinnamate 4-Monooxygenase” or “Vitamin D3 24-Hydroxylase” or “drug metaboli?ing enzyme?” or “Cytochrome Drug-Metaboli?ing Enzyme?” or multigene* or multi-gene*).tw,kf.	163,899
3	1 or 2	190,935
4	(interact* and (“drug-gene” or “drug-drug-gene” or “drug-gene-gene” or EADGI? or “drug-g-gene” or Pharmacogenomic or Pharmaco-genomic? or pharmacogenetic? or pharmaco-genetic? or DDI? or DDGI?)).tw,kf.	10,230
5	(“phenoconversion” or phenoconver* or “pheno?conver*” or ((genotyp* or phenotyp* or extensive* or poor or intermediate or ultrarapid or ultra-rapid or rapid) and metaboli?er*)).tw,kf.	7240
6	“Dose Response”/or “personalized medicine”/or (“precision medicine*” or “personali?ed medicine*” or “Individuali?ed Medicine*” or Theranostic? or “Predictive Medicine*” or “Drug Dose-Response Relationship?” or (treatment* adj2 respon*)).tw,kf.	704,237
7	or/4–6	719,713
8	“Comorbidity”/or (Comorbid* or “Co-morbidity” or Co-morbid* or Multimorbid* or Multi-morbid*).tw,kf.	659,485
9	“Cytokine”/or (“cytokine?” or chemokine?).tw,kf.	779,856
10	“Interleukin 4”/or “Interleukin 6”/or “Interleukin 13”/or (“Interleukin-4” or “B Cell Stimulatory Factor-1” or “B-Cell Growth Factor-1” or “B-Cell Proliferating Factor” or BCGF-1 or Binetrakin or BSF-1 or IL-4 or IL4 or “Mast Cell Growth Factor-2” or MCGF-2 or “Interleukin-6” or “B Cell Stimulatory Factor-2” or “B-Cell Differentiation Factor-2” or BSF-2 or “Hybridoma Growth Factor” or “IFN-beta 2” or “Plasmacytoma Growth Factor” or “Hepatocyte Stimulating Factor” or MGI-2 or “Myeloid Differentiation-Inducing Protein” or “B-Cell Differentiation Factor” or IL-6 or “Interferon beta-2” or IL6 or “Interleukin-13” or IL13 or IL-13).tw,kf.	495,666
11	(“adverse effect?” or “insufficient response?” or “pharmacokinetic?” or (drug adj3 (ineffective* or interaction? or efficac* or safe*)) or “serum concentration?” or polymorphism* or “clinical factor?” or (p?ediatric adj3 (tolerab* or consideration?))).tw,kf.	1,169,207
12	*”oncology”/or neoplasm/or (cancer* or neoplas* or tumo?r* or oncolog*).tw,kf.	5,537,966
13	*”Antineoplastic Agent”/ae, it or (((Antineoplastic or Antitumo?r or Anti-neoplastic or Anti-tumo?r or Anticancer or anti-cancer or Chemotherap* or cancer) adj2 (drug* or therap* or treat* or agent?)) or Antineoplastic? or chemotreat* or chemotherap*).tw,kf.	1,308,484
14	*”Combination Drug Therapy”/ or (“Antineoplastic Combined Chemotherapy Protocol?” or “Combined Antineoplastic Agent?” or “Anticancer Drug Combination?” or “Antineoplastic Combined Chemotherapy Regimen?” or “Antineoplastic Chemotherapy Protocol?” or “Cancer Chemotherapy Protocol?” or “Combination Chemotherap*” or “Drug Polytherap*” or “Combination Drug Therap*” or “Polychemotherap*” or “Poly-chemotherap” or comedicat* or “co-medicat*” or (concomitant adj (medication? or drug?)) or (medication adj manag*) or ((non-competitive or anti-competitive) adj3 inhibition)).tw,kf.	61,154
15	or/8–14	7,970,366
16	3 and 7 and 15	11,583
17	limit 16 to (english language and yr = “2020 − Current”)	2751
18	remove duplicates from 17	2719

**Table A3:** Ovid EBM Reviews—Cochrane Central Register of Controlled Trials May 2024.

#	Searches	Results
1	exp “Cytochrome P-450 Enzyme System”/or Cytochrome P-450 CYP2D6/ge, mt or “Cytochrome P-450 CYP3A”/ge or “Cytochrome P-450 CYP2B6”/ge or “Cytochrome P-450 CYP2C19”/	2286
2	(“Cytochrome P-450*” or “Cytochrome P450*” or “25-Hydroxyvitamin D3 1-alpha-Hydroxylase” or “Aniline Hydroxylase” or “Aromatase” or “Aryl Hydrocarbon Hydroxylases” or “UDP-galactose transporter Benzopyrene Hydroxylase” or “Camphor 5-Monooxygenase” or “Cholestanetriol 26-Monooxygenase” or “Cholesterol 24-Hydroxylase” or “Cholesterol 7-alpha-Hydroxylase” or “Cholesterol Side-Chain Cleavage Enzyme” or “CYP11B2” or “CYP1A1” or “CYP1A2” or “CYP1B1” or “CYP2A6” or “CYP2B1” or “CYP2B6” or “CYP2C19” or “CYP2C9” or “CYP2C8” or “CYP2D6” or “CYP2E1” or “CYP3A” or “CYP4A” or “Limonene Hydroxylases” or “Retinoic Acid 4-Hydroxylase” or “Steroid 11-beta-Hydroxylase” or “Steroid 12-alpha-Hydroxylase” or “Steroid 16-alpha-Hydroxylase” or “Steroid 17-alpha-Hydroxylase” or “Steroid 21-Hydroxylase” or “Steroid Hydroxylases” or “Sterol 14-Demethylase” or “Trans-Cinnamate 4-Monooxygenase” or “Vitamin D3 24-Hydroxylase” or “drug metaboli?ing enzyme?” or “Cytochrome Drug-Metaboli?ing Enzyme?” or multigene* or multi-gene*).tw,kf.	7059
3	1 or 2	7594
4	“Dose-Response Relationship, Drug”/or “Precision Medicine”/or (“precision medicine*” or “personali?ed medicine*” or “Individuali?ed Medicine*” or Theranostic? or “Predictive Medicine*” or “Drug Dose-Response Relationship?” or (treatment* adj2 respon*)).tw,kf.	60,711
5	(interact* and (“drug-gene” or “drug-drug-gene” or “drug-gene-gene” or EADGI? or “drug-g-gene” or Pharmacogenomic or Pharmaco-genomic? or pharmacogenetic? or pharmaco-genetic? or DDI? or DDGI?)).tw,kf.	632
6	(“phenoconversion” or phenoconver* or “pheno?conver*” or ((genotyp* or phenotyp* or extensive* or poor or intermediate or ultrarapid or ultra-rapid or rapid) and metaboli?er*)).tw,kf.	870
7	or/4–6	62,005
8	“Comorbidity”/or (Comorbid* or “Co-morbidity” or Co-morbid* or Multimorbid* or Multi-morbid*).tw,kf.	31,177
9	exp “Cytokines”/or (“cytokine?” or chemokine?).tw,kf.	43,184
10	“Interleukin-4”/or “Interleukin-6”/or “Interleukin-13”/or (“Interleukin-4” or “B Cell Stimulatory Factor-1” or “B-Cell Growth Factor-1” or “B-Cell Proliferating Factor” or BCGF-1 or Binetrakin or BSF-1 or IL-4 or IL4 or “Mast Cell Growth Factor-2” or MCGF-2 or “Interleukin-6” or “B Cell Stimulatory Factor-2” or “B-Cell Differentiation Factor-2” or BSF-2 or “Hybridoma Growth Factor” or “IFN-beta 2” or “Plasmacytoma Growth Factor” or “Hepatocyte Stimulating Factor” or MGI-2 or “Myeloid Differentiation-Inducing Protein” or “B-Cell Differentiation Factor” or IL-6 or “Interferon beta-2” or IL6 or “Interleukin-13” or IL13 or IL-13).tw,kf.	24,218
11	(“adverse effect?” or “insufficient response?” or “pharmacokinetic?” or (drug adj3 (ineffective* or interaction? or efficac* or safe*)) or “serum concentration?” or polymorphism* or “clinical factor?” or (p?ediatric adj3 (tolerab* or consideration?))).tw,kf.	119,196
12	“Antineoplastic Agents”/ae, me or (((Antineoplastic or Antitumo?r or Anti-neoplastic or Anti-tumo?r or Anticancer or anti-cancer or Chemotherap* or cancer) adj2 (drug* or therap* or treat* or agent?)) or Antineoplastic? or chemotreat* or chemotherap*).tw,kf.	95,411
13	“Antineoplastic Combined Chemotherapy Protocols”/or *”Drug Therapy, Combination”/or (“Antineoplastic Combined Chemotherapy Protocol?” or “Combined Antineoplastic Agent?” or “Anticancer Drug Combination?” or “Antineoplastic Combined Chemotherapy Regimen?” or “Antineoplastic Chemotherapy Protocol?” or “Cancer Chemotherapy Protocol?” or “Combination Chemotherap*” or “Drug Polytherap*” or “Combination Drug Therap*” or “Polychemotherap*” or “Poly-chemotherap” or comedicat* or “co-medicat*” or (concomitant adj (medication? or drug?)) or (medication adj manag*) or ((non-competitive or anti-competitive) adj3 inhibition)).tw,kf.	28,658
14	“Medical oncology”/or neoplasms/or (cancer* or neoplas* or tumo?r* or oncolog*).tw,kf.	232,370
15	or/8–14	419,772
16	3 and 7 and 15	957
17	limit 16 to (yr = “2020 − Current” and english language)	161
18	remove duplicates from 17	158

**Table A4:** Clarivate Web of Science Core Collection 2 July 2024.

#	Searches	Results
1	TS = (“Cytochrome P-450*” or “Cytochrome P450*” or “25-Hydroxyvitamin D3 1-alpha-Hydroxylase” or “Aniline Hydroxylase” or “Aromatase” or “Aryl Hydrocarbon Hydroxylases” or “UDP-galactose transporter Benzopyrene Hydroxylase” or “Camphor 5-Monooxygenase” or “Cholestanetriol 26-Monooxygenase” or “Cholesterol 24-Hydroxylase” or “Cholesterol 7-alpha-Hydroxylase” or “Cholesterol Side-Chain Cleavage Enzyme” or “CYP11B2” or “CYP1A1” or “CYP1A2” or “CYP1B1” or “CYP2A6” or “CYP2B1” or “CYP2B6” or “CYP2C19” or “CYP2C9” or “CYP2C8” or “CYP2D6” or “CYP2E1” or “CYP3A” or “CYP4A” or “Limonene Hydroxylases” or “Retinoic Acid 4-Hydroxylase” or “Steroid 11-beta-Hydroxylase” or “Steroid 12-alpha-Hydroxylase” or “Steroid 16-alpha-Hydroxylase” or “Steroid 17-alpha-Hydroxylase” or “Steroid 21-Hydroxylase” or “Steroid Hydroxylases” or “Sterol 14-Demethylase” or “Trans-Cinnamate 4-Monooxygenase” or “Vitamin D3 24-Hydroxylase” or “drug metaboli?ing enzyme?” or “Cytochrome Drug-Metaboli?ing Enzyme?” or multigene* or multi-gene*)	181,166
2	TS = (“precision medicine*” or “personali?ed medicine*” or “Individuali?ed Medicine*” or Theranostic? or “Predictive Medicine*” or “Drug Dose-Response Relationship?” or “treatment* respon*”)	122,486
3	TS = (interact* and (“drug-gene” or “drug-drug-gene” or “drug-gene-gene” or EADGI? or “drug-g-gene” or Pharmacogenomic or Pharmaco-genomic? or pharmacogenetic? or pharmaco-genetic? or DDI? or DDGI?))	6070
4	TS = (“phenoconversion” or phenoconver* or “pheno?conver*” or ((genotyp* or phenotyp* or extensive* or poor or intermediate or ultrarapid or ultra-rapid or rapid) and metaboli?er*))	5330
5	#4 OR #3 OR #2	132,898
6	TS = (Comorbid* or “Co-morbidity” or Co-morbid* or Multimorbid* or Multi-morbid*)	336,994
7	TS = (“cytokine?” or chemokine?)	380,980
8	TS = (“Interleukin-4” or “B Cell Stimulatory Factor-1” or “B-Cell Growth Factor-1” or “B-Cell Proliferating Factor” or BCGF-1 or Binetrakin or BSF-1 or IL-4 or IL4 or “Mast Cell Growth Factor-2” or MCGF-2 or “Interleukin-6” or “B Cell Stimulatory Factor-2” or “B-Cell Differentiation Factor-2” or BSF-2 or “Hybridoma Growth Factor” or “IFN-beta 2” or “Plasmacytoma Growth Factor” or “Hepatocyte Stimulating Factor” or MGI-2 or “Myeloid Differentiation-Inducing Protein” or “B-Cell Differentiation Factor” or IL-6 or “Interferon beta-2” or IL6 or “Interleukin-13” or IL13 or IL-13)	280,695
9	TS = (“adverse effect?” or “insufficient response?” or “pharmacokinetic?” or (drug and (ineffective* or interaction? or efficac* or safe*)) or “serum concentration?” or polymorphism* or “clinical factor?” or (p?ediatric and (tolerab* or consideration?)))	1,475,495
10	TS = (cancer* or neoplas* or tumo?r* or oncolog*)	3,903,978
11	TS = (((Antineoplastic or Antitumo?r or Anti-neoplastic or Anti-tumo?r or Anticancer or anti-cancer or Chemotherap* or cancer) and (drug* or therap* or treat* or agent?)) or Antineoplastic? or chemotreat* or chemotherap*)	2,106,502
12	TS = (“Antineoplastic Combined Chemotherapy Protocol?” or “Combined Antineoplastic Agent?” or “Anticancer Drug Combination?” or “Antineoplastic Combined Chemotherapy Regimen?” or “Antineoplastic Chemotherapy Protocol?” or “Cancer Chemotherapy Protocol?” or “Combination Chemotherap*” or “Drug Polytherap*” or “Combination Drug Therap*” or “Polychemotherap*” or “Poly-chemotherap” or comedicat* or “co-medicat*” or (concomitant and (medication? or drug?)) or (medication and manag*) or ((non-competitive or anti-competitive) and inhibition))	161,321
13	#6 OR #7 OR #8 OR #9 OR #10 OR #11 OR #12	6,150,503
14	#13 AND #5 AND #1	6493
15	#13 AND #5 AND #1 and 2024 or 2023 or 2022 or 2021 or 2020 (Publication Years) and Article (Document Types) and English (Languages)	1259
